# Analysis of SNPs of MC4R, GNB3 and FTO gene polymorphism in obese Saudi subjects

**DOI:** 10.4314/ahs.v17i4.14

**Published:** 2017-12

**Authors:** Said Salama Moselhy, Yasmeen A Alhetari, Archana Iyer, Etimad A Huwait, Maryam A AL-Ghamdi, Shareefa AL-Ghamdi, Khadijah Saeed Balamash, Ashraf A Basuni, Mohamed N Alama, Taha A Kumosani, Soonham Sami Yaghmoor

**Affiliations:** 1 Department of Biochemistry, Faculty of Science, King Abdulaziz University, Jeddah, Kingdom of Saudi Arabia; 2 Department of Biochemistry, Faculty of Medicine, King Abdulaziz University (Rabigh), Kingdom of Saudi Arabia; 3 Consultant cardiologist, King Abdulaziz University hospital, Jeddah, Kingdom of Saudi Arabia; 4 Experimental biochemistry Unit, King Fahd Medical Research Center (KFMRC), KAU, 80216 Jeddah 21589, Saudi Arabia; 5 Production of natural products for industerial health Resaerch Group, King Abdulaziz University, Jeddah, Kingdom of Saudi Arabia; 6 Bioactive natural Research Group, King Abdulaziz University, Jeddah, Kingdom of Saudi Arabia; 7 Department of Biochemistry, Faculty of Science, Ain Shams University, Cairo, Egypt; 8 Department of Biochemistry, Liver Institute, Menofiya University, Egypt

**Keywords:** Obesity, FTO gene-polymorphism

## Abstract

**Background:**

The goal of this study was to analyze the association between the FTO rs17817449 (G>T), G protein beta3 subunit (GNB3) C825T and Melanocortin 4 receptor (MC4R) A822G single nucleotide polymorphism (SNP) with obesity in Saudi subjects.

**Methods:**

The subjects were divided into 2 groups according to BMI: Obese (BMI> 29.9) and non- obese control (BMI<24.9). Genotyping of the target genes were determined by polymerase chain reaction (PCR) followed by restriction fragment length polymorphism analysis (RFLP).

**Results:**

We demonstrated the association of the FTO genotype TT with increased weight, BMI and leptin levels in both males and females. However, there was no association of genotype TT with fasting blood glucose, triglycerides and cholesterol levels. Regarding GNB3 rs5443 polymorphism, the likelihood of obesity was linked to the TT genotype which was also associated with increased leptin levels. On the other hand, the SNP of MC4R A822G did not exhibit any significant association with obesity among studied subjects and showed only the presence of homozygous AA genotype.

**Conclusion:**

The polymorphism of FTO gene rs17817449 and GNB3 gene rs5443 (C825T) may be a genetic determinant of obesity in Saudi population whereas impact of MC4R Asn274Ser change could not be detected.

## Introduction

Increasing prevalence of obesity worldwide prompts many researchers to determine genetic factors underlying this disease. Dina et al.^1^ identified the link between the fat mass and obesity associated (FTO) genotype and obesity among obese European patients. Moreover, the FTO genotype has been reported to be associated with phenotypic variability of BMI^2^. In parallel, the heterotrimeric G proteins, which are key components of intracellular signal transduction and play a focal role in adipogenesis, have been proposed as candidate genes for obesity^3^. C825T polymorphism in the G protein beta^3^ subunit (GNB3) showed to play an important role in the determination of obesity in the German population^4^. In addition, Melanocortin 4 receptor (MC4R) deficiency that resulted from disruption of one or both MC4R alleles represents the commonest monogenic form of human obesity to date^5^. Of note, frequency of MC4R gene mutations was found to be lower in some studies than others, accounting for ∼ 6% of severe obesity cases^6^–^8^. However a significance of MC4R mutations in Asian obese populations has not been adequately detected compared with non-obese^9^–^11^.

In Saudi Arabia, which has undergone significant economic and cultural changes over the past thirty years, the prevalence of obesity has increased dramatically especially among women and showed to be 23.6% versus 14.2% among men^12^. The significance of FTO rs17817449, GNB3 rs5443 and MC4R gene mutation in Saudi obese populations has not been examined despite its association with obesity in other parts of the world. The aim of this study was to evaluate the association of the FTO rs17817449 (G>T), GNB3 rs5443 (C825T) and MC4R Asn274Ser (A822G) gene polymorphism with obesity and obesity-related metabolic traits in Saudi subjects.

## Subjects and methods

Two hundred and twelve unrelated individuals were included in this study, 107 males and 105 females, with mean age of 30.74+10.76 and 35.64+11.01 respectively. The subjects were divided into 2 groups according to BMI; obese (BMI ≥ 30 kg/m^2^), included 51 males and 55 females, and non - obese (BMI<24.9), included 56 males and 50 females. The subjects were recruited from November 2010 to Jun 2012 at King Fahd Medical Research Center (KFMRC), Mada'en Al-Fahad Medical Center and Medical administration in King Abdulaziz University. This study was approved by the ethics committee of the King Abdul-Aziz University Hospital, Jeddah, Saudi Arabia (reference No 741-12) for sample collection. Written informed consent was obtained from all participants prior to the study.

After an overnight fast, blood samples were withdrawn in the morning from all subjects and divided into two parts. The first was transferred into EDTA containing tubes for DNA extraction and genotyping of studied genes. The second part was transferred into a dry sterile tube and allowed to clot. The serum was separated and divided into aliquots for biochemical and hormonal analysis. They were kept frozen at −80°C for further analysis.

Biochemical parameters such as fasting glucose, total cholesterol, triglyceride, and HDL-C were determined, by timed endpoint method, using commercially available test kits (Roche Diagnostics, Mannheim, Germany). LDL-C was calculated by the Friedewald formula.

### Serum hormones were measured by ALPCO immunoassays kit (Human leptin Alpco, USA).

Genetic analyses and genomic DNA was extracted from whole blood in EDTA tubes using a standard salting-out. For FTO rs17817449, briefly, 5′-GGTGAAGAGGAGGAGATTGTGTAACTGG-3′ and 5′-GAAGCCCGTAGAAGTTTAGAGTAAATTGGG -3′ primers were used for amplification followed by restriction analysis with AlwNI enzyme (Fermentas, Lithuania) according to Hubacek et al.^13^. The uncut PCR product of 198 bp represents allele G, while restriction fragments of 99 bp represent allele T.

While for determination of GNB3 C825T allele, 5′-TGACCCACTTGCCACCCGTGC-3′ and -3′ -GCAGCAGCCAGGGCTGGC-5′ primers were used for amplification followed by cutting with BsaJ1 restriction enzyme (Biolabs, USA) according to Ohshiro et al.^14^, to determine the GNB3 C825T polymorphism. Alleles T represent the absence of restriction site (268-bp) while alleles C indicate the presence of restriction site (152-bp and 116-bp bands).

The PCR thermal cycle include different temperature (94 −95°C), denatures the double stranded DNA, 60 −72°C , annealing of primer 72°C DNA extension polymerase. MC4R gene (A822G) polymorphism was detected by a PCR- restriction fragment length polymorphism according to Yurtcu et al.^15^.

## Statistical analysis

The p value at <0.05 was considered as significant. Deviation from Hardy-Weinberg equilibrium for FTO genotypes and GNB3 genotypes was calculated by the Chi-square test.

## Results

**Figure(1 a,b):**
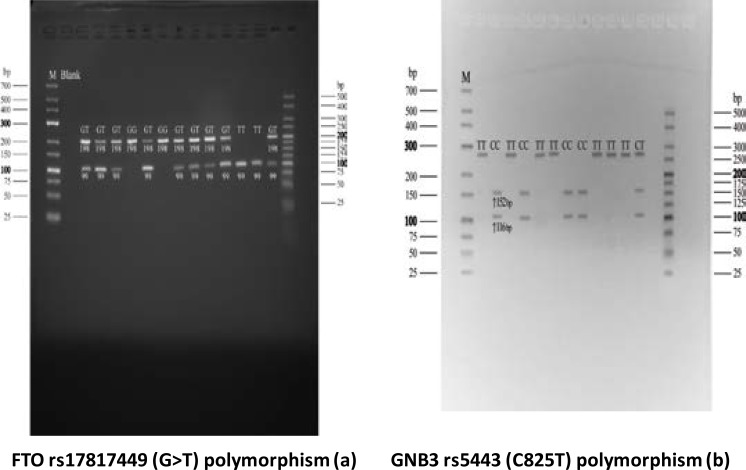
Analysis of FTO rs17817449, GNB3 rs5443 and MC4R gene Polymorphism

Analysis of FTO rs17817449, GNB3 rs5443 (C825T) and MC4R A822G polymorphism are showed in figure 1. In FTO polymorphism, the G allele generated an undigested 198-bP product while the T allele yielded 99-bp fragment after digestion. While in GNB3 C825T polymorphism, alleles T represent the absence of restriction site, giving a 268-bp PCR product, and alleles C indicate the presence of restriction site giving 152-bp and 116-bp fragments. For MC4R gene (A822G) polymorphism, the uncut PCR product of 382 bp represents allele G, while restriction fragments of 45bp and 337bp represent allele A.

**Figure (2 a,b):**
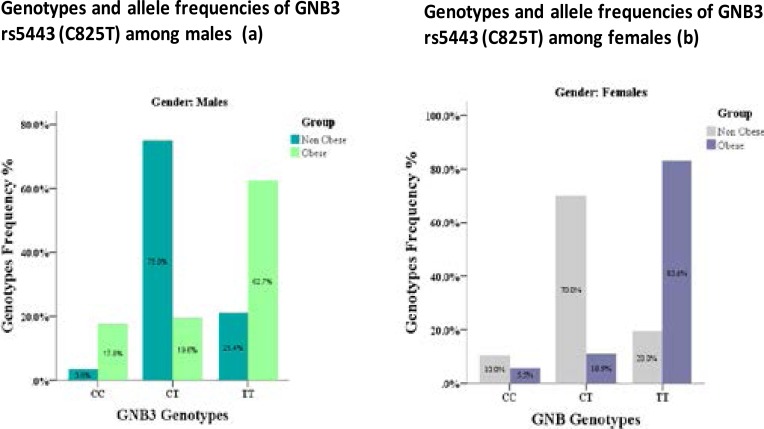
Genotypes and allele frequencies of FTO rs17817449 (G>T) and GNB3 rs5443 (C825T) among obese and non obese subjects

Figure 2, shows the genotype frequencies of both FTO rs17817449 (G>T) and GNB3 rs5443 (C825T) polymorphism. In males, the genotyping of FTO rs17817449 (G>T) was as follows; homozygous GG (%25.5), heterozygous GT (43.1%), and homozygous TT (31.4%) in obese group versus GG (23.2%), GT (73.2 %) and TT (3.6 %) respectively in non-obese group. In females, the frequencies were GG (n= 10; 18.2%), GT (n= 29; 52.7%) and TT (29.1%) in obese group versus GG (16.0%), GT (80.0%) and TT (4.0%) respectively in non-obese group. Genotype frequencies of GNB3 rs5443 (C825T) polymorphism in males subjects were as follows; homozygous CC (n=9; 17.6%), heterozygous CT (19.6%), and homozygous TT (n=32; 62.7%) in obese group versus CC (3.6 %), GT (75.0%) and TT (n=12; 21.4%) respectively in non-obese group. In females the frequencies were CC(5.5%), CT (10.9%) and TT (83.6%) in obese group versus GG (10%), GT (70%) and TT (20%) respectively in non-obese group.

The allele frequencies of both FTO rs17817449 (G>T) and GNB3 rs5443 (C825T) are showed in table 2.

**Table 2:** Allele frequencies of FTO rs17817449 (G>T) and GNB3 rs5443 (C825T) Polymorphism among obese and non obese subjects (males and females).

Sex and Allele		Frequencies %		*P*-value	Odd Ratio (95%CI)	Risk Ratio (95%CI)
		Non obese	Obese			
**FTO rs17817449 (G>T)**						
**TT+GT**		76.8% (n=43)	74.5% (n=38)	0.777	0.88 (0.37–2.14)	0.97 (0.78–1.20)
**Male**	**G**	59.8% (n=67)	47.1% (n=48)	0.043^a^	1.75 (1.02–3.03)	1.35 (1.00–1.82)
	**T**	38.4% (n=43)	52.9% (n=54)			
**Female**	**G**	56.0% (n=56)	44.5% (n=49)	0.097	1.58 (0.92–2.73)	1.26 (0.95–1.66)
	**T**	44.0% (n=44)	55.5% (n=61)			
**GNB3 rs5443 (C825T)**						
**CC+CT**		78.6% (n=44)	37.2% (n=19)	0.001^c^	0.096 (0.02–0.49)	0.26 (0.07–0.92)
**Male**	**C**	41.1% (n=46)	27.5% (n=28)	0.036^b^	1.84 (1.04–3.26)	1.23 (1.01–1.50)
	**T**	58.9% (n=66)	72.5% (n=74)			
**Female**	**C**	45% (n=45)	10.91% (n=12)	<0.0001^b^	6.68 (3.26–13.69)	1.62 (1.34–1.96)
	**T**	55% (n=55)	89.09% (n=98)			

a G *vs.* T

b C *vs.* T, *P*-value Person Chi-Square test

c CC *vs.* CC+CT, *P*-value Fisher Exact test

Comparative analysis of the allelic frequencies of FTO rs17817449 polymorphism in obese and non-obese groups revealed a significant difference in males (P=0.043) and non significant difference in females (P=0.097). The frequency of the G allele was more pronounced among non obese, males and females, 59.8% and 56.0%, than obese subjects, 47.1% and 44.5% respectively. On the other hand, T allele was more pronounced among obese males and females, 52.9% and 55.5% than non-obese 38.4% and 44.0% respectively. The odd ratio between G allele and T allele in males and females was 1.75 and 1.58 respectively. There was a statistically significant relationship between T allele and obesity.

The association of FTO rs17817449 (G>T) genotypes with obesity and metabolic related traits are showed in Table 3. Both TT and GG genotypes had a higher weight and BMI values than that found in heterogeneous genotype GT. Also, the TT showed a higher weight and BMI values than that found in genotype GG, which was significant among males and only significant regarding BMI among females. HDL-C levels were lower in genotype TT in comparison with those found in genotypes GG and GT. This difference was only significant among male subjects between TT and GG genotypes.

**Table 3:** FTO genotype frequencies, anthropometric, biochemical and hormonal parameters in male and female subjects

Variables	Frequency %					
	GG M	F	GT M	F	TT M	F
	*(n=26)* *24.3%*	*(n=18)* *17.1%*	*(n=63)* *58.9%*	*(n=69)* *65.7%*	*(n=18)* *16.8%*	*(n=18)* *17.1%*
**Weight**	91.52±30.99	84.88±28.01	79.80±23.90^a^	69.41±18.64^a^	112.26±31.10^b,c^	94.66±27.33 ^c^
**BMI**	30.86±9.24	33.38±10.76	27.74±8.29 ^a^	27.33±6.44^a^	38.55±8.73^b,c^	36.30±9.77 ^b,c^
**Glucose**	104.96±29.00	111.27±41.66	107.34±48.47	108.69±50.80	116.38±61.54	111.76±23.29
**Cholesterol**	178.50±39.12	190.27±35.29	176.85±38.37	198.56±40.31	179.22±52.29	214.33±52.98
**Triglycerides**	106.30±55.28	105.27±78.66	117.25±93.94	115.11±78.41	123.38±61.55	131.61±98.16
**HDL-C**	43.03±9.61	48.22±10.90	40.73±9.27	53.66±13.12	37.20±7.83^b^	47.72±11.88
**LDL-C**	120.98±33.63	121.0±28.94	119.54±40.24	121.74±32.55	103.46±24.86	137.09±41.82
**Leptin Hormone**	30.34±26.01	60.83±33.77	20.58±22.52	43.03±29.49^a^	40.68±25.26^c^	64.81±36.32^c^
**Growth Hormone**	0.295±0.609	0.671±0.853	0.374±0.77	1.16±1.96	0.124±0.111^c^	0.667±0.686
**C-Peptide**	0.850±0.249	0.958±0.262	0.823±0.44	0.704±0.307^a^	0.957±0.537	1.018±0.675

Results are expressed as mean± SD, and were compared by t-test (*P* < 0.05).

*P*-value^a^: GG vs. GT

*P*-value^b^: GG vs. TT

*P*-value^c^: GT vs. TT

Regarding the hormones, both TT and GG genotypes showed to have significant higher leptin levels than that found in GT genotypes among females; while in males, this difference was only significant between TT and GT genotypes. A similar association was found with C peptide, where genotypes TT and GG had higher C peptide levels compared to that found in GT genotype among males or females, with only statistical difference between GG and GT among females.

On the other hand, the growth hormone (GH) levels were lower in genotypes TT and GG than in genotype GT. This difference was only significant between TT and GT genotypes among males. The c-peptide is a good index for insulin secretion and clinically related to metabolic syndrome and diabetes.

In table (4) the genotype TT and CC were associated with higher significant weight and BMI than that found in genotype CT among males, while among females the difference was only significant between TT and CT genotypes. Regarding the hormones, the TT genotype showed higher leptin levels than that found in CT or CC genotype among males and females. This difference was only significant between TT and CT genotypes. A similar association was found with C peptide, where genotype TT showed higher C peptide levels than that found in CT and CC genotypes. To the contrary, the growth hormone (GH) levels were significantly lower in genotype TT compared with that of genotype CT in males and females but not statistically different from that found in CC genotype.

**Table 4:** G-protein β3 subunit gene frequencies, anthropometric, biochemical and hormonal parameters in male and female subjects

Variables	Frequency %					
	CC M	F	CT M	F	TT M	F
	*(n=11 )* *10.3%*	*(n=8 )* *7.6%*	*(n=52 )* *48.6*	*(n=41 )* *39.0%*	*(n= 44)* *41.1%*	*(n= 56)* *53.3%*
**Weight**	106.6±27.93	79.92±25.71	77.35±26.76	64.73±22.98	99.19±29.04^C^	84.43±21.44^c^
**BMI**	33.49±6.68	30.57±10.89	26.12±7.70	25.52±7.76	34.30±9.58^C^	33.02±7.68^c^
**Glucose**	122.81±51.14	94.12±17.20	102.88±44.07	96.17±21.64	111.0±49.26	121.48±56.45^bc^
**Cholesterol**	195.63±47.33	180.0±28.60	187.80±34.00	198.56±40.31	194.0±53.96^C^	211.50±45.98^bc^
**Triglycerides**	103.27±59.93	83.87±41.43	96.34±74.75	92.43±64.35	141.5±86.28^C^	138.32±91.16^bc^
**HDL-C**	39.20±10.51	56.03±19.03	41.78±9.40	52.22±9.70	39.79±8.78	51.19±13.91
**LDL-C**	135.70±44.99	107.18±23.93	109.76±30.49	118.06±26.99	120.58±40.85^C^	131.91±38.04^bc^
**Leptin** **Hormone**	27.90±21.57	51.48±36.30	21.57±27.42	37.35±27.65	31.60±21.69^c^	58.71±32.92^c^
**Growth** **Hormone**	0.142±0.193	1.81±2.98	0.511±0.912^a^	1.37±1.99	0.145±0.268^c^	0.609±0.983^c^
**C-Peptide**	0.941±0.247	0.728±0.338	0.740±0.390	0.802±0.445	0.969±0441^c^	0.811±0.398

## Discussion

The present study showed that serum leptin level increases significantly as the BMI increases (table 1). Similar results have been reported by previous studies where leptin concentrations showed to be correlated in healthy individuals with the body fat content and body mass index^18^ Several factors have been proposed for such increase of leptin levels like; a diminished response in the leptin receptor signalling pathway, poor penetration of the bloodbrain barrier by leptin, the presence of less active molecular forms of leptin or Leptin resistance^19^.

**Table 1:** Anthropometric parameters and serum levels of biochemical and hormonal parameters in male and female groups

Variables	Males		P-Value	Females		P-Value
	Non Obese (n= 56)	Obese (n=51)		Non Obese (n=50)	Obese (n=55)	
**Weight (KG)**	65.73±6.97	115.26±23.44	0.000	57.45±5.82	93.61±21.27	0.000
**BMI**	22.77±2.06	38.48±6.69	0.000	22.79±2.05	36.37±7.19	0.000
**Glucose**	97.98±26.72	119.70±56.36	0.014	94.67±24.39	122.98±54.99	0.001
**Cholesterol**	173.71±37.62	193.03±51.60	0.028	185.34±34.93	213.03±44.11	0.001
**Triglycerides**	97.00±78.19	136.07±79 .30	0.012	85.92±62.27	143.83±87.74	0.000
**HDL-C**	42.58±9.51	38.63±8.55	0.026	54.91±18.77	49.85±12.08	0.048
**LDL-C**	111.23±35.29	128.68±40.49	0.019	114.05±29.73	134.23±34.93	0.002
**Serum leptin** **Hormone**	11.01±11.80	42.98±24.63	0.000	28.38±14.35	69.31±32.28	0.000
**Growth** **Hormone**	0.48±0.87	0.191±0.434	0.038	1.72±2.24	0.42±0.58	0.000
**Fasting** **serum C-Peptide**	0.713±0.348	1.00±0.427	0.000	0.645±0.198	0.936±0.491	0.000

It has long been known that C-peptide levels in the blood and urine provide an accurate estimate of insulin secretion rates. The association between C-peptide levels with diabetes severity and obesity has been hypothesized in previous studies^20^–^23^. In the current study, the fasting C-peptide levels were found to be significantly higher in obese group in comparison with that found in non-obese group (table 1). Our study results and those by others, Pasquali et al.^24^, Park et al.^25^, suggested that obese subjects are hyperinsulinemic.

In the current study, we explored the association of FTO rs17817449 (G>T), GNB3 rs5443 (C825T) and MCR4 (A822G) genes SNP with obesity and obesity-related metabolic traits in a small sample of Saudi population.

The FTO gene is highly polymorphic, and several polymorphisms of the gene have been found to be associated with obesity or obesity phenotypes^26^–^30^. Among such polymorphisms, the FTO rs17817449 gene SNP showed to be associated with obesity in several populations^30^–^32^. In the present study, the association of FTO genotype TT with higher BMI was stronger than that of genotype GG. Although this seems contradictory to several earlier reports, it is still possible and can be explained by the much lower frequency of FTO rs17817449 in Asian and African populations than those in white populations, showing a smaller effect than that detected in Europeans^31^.

In parallel, fried food consumption and particularly saturated fatty acids, seemed to determine or modulate the association between the FTO risk-allele and higher BMI^30^. Obviously, the role of genetic variation at the FTO locus in predisposing to obesity in Asian, Saudi, populations warrants further investigation especially in relation to the epidemiological transition and access to a calorie-rich diet.

In the current study, we did not find any association between FTO rs17817449 SNP and fasting glucose. This is in line with the findings of Hubacek et al.^19^ FTO was first identified as a type 2 diabetes susceptibility gene, but, as further adjustment for BMI abolished the association with diabetes^32^, it was suggested that FTO is primarily an obesity-susceptibility locus.

In this study, we showed that the FTO rs17817449 was associated with higher leptin concentrations regardless of gender. In parallel, the association of the FTO rs9939609 polymorphism with serum leptin concentrations showed association between the A allele and serum leptin levels, but it was not adjusted for BMI and was considered as a result of increased adiposity^28^.

In the present study, the TT genotype was associated with a decrease of GH in comparison with GT genotype. This association was found among males when stratified for gender. The association of GH deficiency with obesity in humans and determining whether or not FTO regulates GH and/ or other hormones secreted by the hypothalamus-pituitary axis will greatly elucidate the FTO's physiological function in future^22^. Regarding FTO genotype and C-peptide, the association was statistically significant between GG and GT genotypes in the total group and female gender. It was reported that, there was no significant association between the FTO rs17817449 SNP and C-peptide in male gender.

In this study, the GNB3 TT genotype was more frequent in obese subjects, males or females, the frequency was (62.7% and 83.6%) versus (21.4% and 20%) respectively, in non-obese subjects suggesting the possible relation with obesity. In parallel, we demonstrated a significant association of the TT genotype with higher levels of weight and BMI compared with CT genotype. The T allele of GNB3 rs5443 SNP has been reported to predispose to obesity in German, Chinese and South African populations.

In the current study, we investigated Asn274Ser non-synonymous mutation of the MC4R gene that has been linked to obesity in previous studies^21^. We only detected the presence of homozygous AA genotype while AG and GG were not observed and MC4R gene SNP did not exhibit any significant association with obesity among studied subjects. However, a positive association between Asn274Ser mutation and obesity in Turkish population.

## Conclusion

The polymorphism of FTO gene rs17817449 and GNB3 gene rs5443 (C825T) may be a genetic determinant of obesity in Saudi population, whereas impact of MC4R Asn274Ser change could not be detected in our sample.
